# Bioactive cellulose acetate nanofiber loaded with annatto support skeletal muscle cell attachment and proliferation

**DOI:** 10.3389/fbioe.2023.1116917

**Published:** 2023-02-23

**Authors:** Ana Elisa Antunes dos Santos, Tiago Cotta, João Paulo Ferreira Santos, Juliana Sofia Fonseca Camargos, Ana Carolina Correia do Carmo, Erika Gabriele Alves Alcântara, Claudia Fleck, Aline Gonçalves Lio Copola, Júlia Meireles Nogueira, Gerluza Aparecida Borges Silva, Luciana de Oliveira Andrade, Roberta Viana Ferreira, Erika Cristina Jorge

**Affiliations:** ^1^ Departamento de Morfologia, Instituto de Ciências Biológicas, Universidade Federal de Minas Gerais, Belo Horizonte, Minas Gerais, Brazil; ^2^ Departamento de Engenharia de Materiais, Centro Federal de Educação Tecnológica de Minas Gerais (CEFET-MG), Belo Horizonte, Brazil; ^3^ Technische Universität Berlin, Chair of Materials Science and Engineering, Berlin, Germany

**Keywords:** cultivated meat, muscle tissue engineering, cellulose acetate, annatto, nanofiber, scaffold, electrospinning

## Abstract

Electrospinning emerged as a promising technique to produce scaffolds for cultivated meat in function of its simplicity, versatility, cost-effectiveness, and scalability. Cellulose acetate (CA) is a biocompatible and low-cost material that support cell adhesion and proliferation. Here we investigated CA nanofibers, associated or not with a bioactive annatto extract (CA@A), a food-dye, as potential scaffolds for cultivated meat and muscle tissue engineering. The obtained CA nanofibers were evaluated concerning its physicochemical, morphological, mechanical and biological traits. UV-vis spectroscopy and contact angle measurements confirmed the annatto extract incorporation into the CA nanofibers and the surface wettability of both scaffolds, respectively. SEM images revealed that the scaffolds are porous, containing fibers with no specific alignment. Compared with the pure CA nanofibers, CA@A nanofibers showed increased fiber diameter (420 ± 212 nm vs. 284 ± 130 nm). Mechanical properties revealed that the annatto extract induces a reduction of the stiffness of the scaffold. Molecular analyses revealed that while CA scaffold favored C2C12 myoblast differentiation, the annatto-loaded CA scaffold favored a proliferative state of these cells. These results suggest that the combination of cellulose acetate fibers loaded with annatto extract may be an interesting economical alternative for support long-term muscle cells culture with potential application as scaffold for cultivated meat and muscle tissue engineering.

## Introduction

Cultivated meat is an alternative source of animal protein for the increasing meat demand, explored to overcome the known problems of the livestock production methods, such as environmental impact, animal welfare and public health (Post, 2012; Bomkamp et al., 2021). It is meat produced by tissue engineering technique, which corresponds to the *in vitro* cultivation of myogenic cell lineages or muscle stem cells in a scaffold, capable of mimicking the extracellular matrix of the tissue (Ben-Ayre and Levenberg, 2019; Choi et al., 2020; Ahmad et al., 2021).

Besides mimicking the extracellular matrix, scaffolds used to produce cultivated meat need to support the development, growth and differentiation of the myogenic lineage in a mature muscle tissue in *vitro* culture conditions (Howard et al., 2008; Xing et al., 2019). In order to develop efficient scaffolds for cultivated meat, it is essential to take into account the structure of naturally occurring muscle tissue. Muscle tissue is composed of multinucleated cells (myofibers) that are uniaxially aligned along the main axis of the tissue (Pardo et al., 2021). Myofibers are long cells, with ∼40 mm in length and 10–100 μm in diameter, composed of bundles of contractile filaments composed of long chains of actin and myosin (myofibrils), 1–3 μm in size (Jun et al., 2009). It is also known that skeletal muscle exhibits anisotropic mechanical and electrical responses. Therefore, creating anisotropic scaffolds with micro- or nanoscale properties has become a common strategy for building muscle tissue engineering constructs (Grasman et al., 2015; Smoak and Mikos, 2020; Pardo et al., 2021). Approaches using micropatterning techniques to regulate cell alignment have been found to be effective in mimicking muscle tissue structure, composition, and function (Nakamoto et al., 2014; Xiang et al., 2022). Such materials have demonstrated the ability to induce muscle cell alignment, promote myogenic differentiation at early stages for cell fusion, and develop long and thick myotubes due to their morphological and topographical characteristics (Liu et al., 2017; Vogt et al., 2017; Bloise et al., 2018; Narayanan et al., 2020).

However, muscle tissue is composed of both aligned extracellular matrix (ECM) fibrils and a random mesh of collagen fibrils of connective tissues. Connective tissues, such as endomysium, perimysium, and epimysium, provide force transmission and mechanical support to the muscle architecture and are composed of a strong collagen network (Uehara et al., 2020). Most of the load capacity of muscle arises from the dense ECM that forms these tissues rather than the aligned muscle fibers, revealing the importance of a strong support structure to sustain mature muscle cells. Therefore, recapitulating the mechanical properties of the muscle tissue by using scaffolds mechanically similar to the ECM is essential for cultured meat to achieve the texture of conventional meat. It is also extremely important that the scaffolds are easy to manipulate and can resist the forces applied during the processing (Bookamp et al., 2021). A hybrid combining aligned and random nanofibers were presented by Park et al. (2016). While the aligned fibers provided uniaxial topographic guidance for muscle cell development, the random fibers enhanced mechanical stability, support, and adequate permeability, and were amenable to physical manipulation. Scaffolds can also be used in combination with bioactive cues, such as growth factors, ECM proteins, or cell-signaling peptides, to promote specific cell behaviors (Borselli et al., 2011; Cezar and Mooney, 2015). Additional strategies to influence skeletal muscle cell differentiation and growth in scaffolds include mechanical and electromagnetic stimuli. Mechanical passive, phasic, and gradual stretch stimuli have been applied in cell-laden gel constructs to promote myotubes alignment and growth (Nishiguchi et al., 2011; Simsa et al., 2019; Kang et al., 2021). Electrical stimulation also improved the maturation of bovine myotubes and C2C12 myoblasts cultured in aligned scaffolds (MacQueen et al., 2019; Orellana et al., 2020). In addition, cell culture on conductive biomaterials such as polyaniline (Jun et al., 2009), gold or titanium coatings (Yang et al., 2016), or in the presence of magnetic nanoparticles and under external magnetic field stimulation (Pardo et al., 2022), has been reported as a strategy to enhance myotube maturation.

Electrospun nanofiber scaffolds present an interesting alternative for muscle cell cultivation because they can better simulate typical muscle fibrous architecture. Its nanoscale structure mimics the extracellular matrix and induces great cellular attachment due its nanofiber high aspect ratio, porosity and surface-to-volume ratio (Hejazian et al., 2012). Previous studies have demonstrated the importance of the nanoscale structure and its anisotropy in synthetic polymers for the development of 3D matrices (Mitchell & Tojeira, 2013; Marzio et al., 2020). Nanofibers contribute to rapid diffusion of oxygen and nutrients, as well as cell infiltration, promoting better cell proliferation and biocompatibility (Perez-Peruvyan et al., 2021). In addition, nanofiber scaffolds have the ability to induce cell alignment along the fibers that might induce muscle fiber maturation (Baker and Mauck, 2007).

Cellulose-based biomaterials offer some important advantages over conventional synthetic materials and show great scientific promise (Hickey and Pelling, 2019). Several studies have demonstrated that the hydrophilic hydroxyl moieties of the cellulose and specialized cellulose binding domains provide sites that favor adhesion and proliferation (Elsayed et al., 2020; Marino et al., 2021). Cellulose acetate (CA) is a modified natural polymer that has good solubility and mechanical properties, demonstrates biodegradability and biocompatibility, and can be easily controlled morphologically (Liu and Hsieh, 2002; Bifari et al., 2016; Angel et al., 2020). In addition, CA shows good fiber-forming ability, or spinnability, using a variety of solvents (Konwarh et al., 2013; Sánchez-Cid et al., 2022).

CA nanofibers are very interesting in cultivated meat applications because, in addition to being a low-cost material, their fabrication by the electrospinning process is relatively easy (Angel et al., 2020; Marino et al., 2021). Besides, contrary to scaffolds composed of plant-based materials, they do not need to be coated with ECM proteins or chemical modification to improve cell adhesion (Hickey and Pelling, 2019; Xiang et al., 2022). Santos et al. (2021) demonstrated that it is possible to grow fibroblasts on CA nanofibers without the need for coating. Thus, the application of CA nanofibers in a cultured meat production process may be a more economical option compared to other synthetic polymers. CA nanofiber incorporated into chitosan/silk fibroin scaffold has improved the proliferation, infiltration, and contractility of smooth muscle cells (Zhao et al., 2022). Nevertheless, studies with CA nanofibers for applications in tissue engineering and cultured meat are still scarce.

Although various nanofiber scaffolds have been developed for biomedical applications, few investigations have been done for applications in cultivated meat (Allen et al., 2017; MacQueen et al., 2019; Zoldan and Allen, 2019). MacQueen et al. (2019) demonstrated the growth of rabbit and bovine smooth muscle cells on rotary jet spun gelatin as well as a histological comparison of the engineered constructs to rabbit muscle, bacon, and ground beef. PCL and PNIPAAm scaffolds have been used to produce aligned cell sheets *via* electrospinning for application in muscle cell cultivation (Allen et al., 2017). The technique was patented by the cultivated meat company BioBQ for the potential development of cultivated jerky and brisket beef (Zoldan and Allen, 2019). In addition, edible and biodegradable electrospun nanofiber has been developed by cultivated meat companies such as Matrix Meats and Gelatex (Bomkamp et al., 2021).

Another important point in food production is preservation, which nowadays is focused on the use of natural products (Hernández-Ochoa et al., 2014). Recently, essential oils extracted from plants have received a lot of attention due to their meat protection properties. Antimicrobial properties of plant essential oils are derived from some main bioactive components such as phenolic acids, terpenes, aldehydes, and flavonoids (Patra, 2012). Various mechanisms such as changing the fatty acid profile and structure of cell membranes and increasing the cell permeability as well as affecting membrane proteins and inhibition of functional properties of the cell wall are effective in antimicrobial activity of essential oils (Yousefi et al., 2020). Annatto is the fruit of the annatto (*Bixa orellanna L.*) native to South America. Annatto seeds are considered antibiotics of medicinal character, acting as an anti-inflammatory for bruises and wounds, also having been used in the cure of bronchitis and external burns. In addition, annatto has a long history of use in the food industry as a natural dye (Cardarelli et al., 2008; Rivera-Madrid et al., 2016; Shahid-ul-Islam et al., 2016). Our research group produced scaffolds from cellulose acetate nanofibers loaded with annatto extract and demonstrated that the scaffold maintained the viability of mouse fibroblasts after 48 h of culture, in addition to allow cell attachment, spreading and colonization of the nanofiber (Santos et al., 2021).

Here we investigated physicochemical, morphological, mechanical and biological features of bioactive cellulose acetate (CA) and cellulose acetate loaded with annatto extract (CA@A) nanofibers to evaluate their potential for application in cultivated meat. We found that cellulose acetate nanofibers loaded with annatto extract favored cell adhesion and improved cell viability and long-term cell proliferation. Furthermore, random CA nanofiber favored the myoblast differentiation profile.

## Materials and methods

### Cellulose acetate (CA) and cellulose acetate with annatto extract (CA@A) nanofibers physicochemical characterization

The cellulose acetate (CA) and cellulose acetate with annatto extract (CA@A) nanofibers were obtained by electrospinning, as previously described (Santos et al., 2021). Briefly, crude annatto extract was obtained using the solvent extraction method. Annatto seeds were washed with distilled water to remove any adhering powder, and then macerated using a ceramic mortar and pestle. Macerated seeds were soaked in 0.05 g/mL ethanol. The mixture was stirred magnetically at 50°C for 60 min, and then filtered through a Whatman filter. The polymer was impregnated with the crude extract by mixing 5 g of powdered cellulose acetate with 20 mL crude annatto extract. The homogeneous mixture was then placed under a fume hood at room temperature (RT) for the ethanol to evaporate, and subsequently kiln dried at 50°C for 30 min. The cellulose acetate nanofibers (CA) and cellulose acetate with annatto extract nanofibers (CA@A) were obtained by electrospinning as described below: the cellulose acetate and cellulose acetate with crude annatto extract were dissolved in acetone-dimethylformamide (3:1 v/v) to obtain 12 wt% (w/v) solution. The polymer solution was fed into a 10 mL standard syringe attached to a 0.3 mm (gauge 30) inner diameter stainless needle. The electrospinning process utilized electric voltage of 12 kV, 10 cm working distance, collector rotation at 200 rpm, and 0.8 mL/h solution feed rate at room temperature (NB-EN1, NanoBond). Physicochemical characterization of the nanofibers was performed using the following analysis: i) UV-vis spectroscopy; ii) contact angle analyzer; and iii) Nanoscale Dynamic Mechanical Analysis.

The UV-vis spectroscopy of annatto extract was performed in a Perkin Elmer Lambda 1,050 spectrometer (Waltham, USA), with wavelength range of 250–800 nm and scanning speed of 267 nm/min. The annatto extract used in this analysis was diluted in acetone 1:50 (v/v) and the measurements were obtained right after its preparation. CA and CA@A nanofibers were also evaluated to determine their surface wettability, which was measured using a contact angle analyzer (KRÜSS model DSA-100; KRÜSS Scientific, Hamburg, Germany). Deionized water was automatically dripped onto the nanofiber samples and five contact angle measurements were averaged to obtain a reliable value.

Nanoscale Dynamic Mechanical Analysis (Nano-DMA) was performed using the Hysitron TI950 TriboIndenter device (Bruker Corporation, Billerica, USA) equipped with a Berkovich tip. Nanofiber samples were glued to an epoxy holder to ensure stability during measurement. A grid with 100 measurement points (10 × 10) was created for oscillatory measurements to simultaneously obtain both the linear- and visco-elastic responses of the sample. Specimens were loaded with a sinusoidal force-time-function and a maximum load of 75 μN oscillating at eight different frequencies (10, 31, 25, 115, 136, 157, 178, and 201 Hz). Loss (E″) and storage modulus (E’) were calculated from the measured force-displacement hysteresis loops using the software provided with the Bruker nanoindenter. The indents are approximately twice as small as the fiber diameter. We assumed that if an indent reached a pore, it would measure the fiber directly below it.

### Morphological characterization of the nanofibers

CA and CA@A nanofibers were also morphologically characterized using the Phenom XL (Phenom-World, Eindhoven, Netherlands) scanning electron microscope (SEM), with medium vacuum (60 Pa) and auto focus on an accelerating voltage of 5 kV. Nanofiber samples were sputtered with gold for 20 min, using a sputter coater (Cressington 108 model, Cressington Scientific Instruments).

Next, SEM images were used to obtain the average of fiber diameter, using the ImageJ software. From three SEM images from each nanofiber sample, 200 randomly selected fibers were measured using the line tool of the ImageJ software.

### C2C12 cell culture

Immortalized mouse myoblasts from the C2C12 cell lineage (ATCC^®^ CRL1772™) were used in this work. C2C12 cells were maintained in growth medium [GM: DMEM-high glucose (Gibco), supplemented with 10% bovine fetal serum (Gibco) and 1% anti-anti (Gibco)], at 37°C and 5% CO_2_. Cells were used among the fourth and eighth passages. When applicable, cell differentiation was induced at low serum condition [DM: DMEM (Gibco), supplemented with 2% Horse Serum (Gibco) and 1% anti-anti (Gibco)].

### C2C12 cell seeding onto CA and CA@A nanofibers

CA and CA@A nanofibers were sterilized using gamma irradiation, at RT with a standard dose of 10 kGy. ^60^Co gamma-ray source was used. Gamma irradiation sterilization was carried out at Gamma Irradiation Laboratory installed at the Nuclear Technology Development Centre (CDTN, Belo Horizonte, Brazil).

Before cell seeding, CA and CA@A nanofibers were cut into 16 mm disks and fixed in the well of a 24-well plate. The disks were equilibrated using 200 μL of GM for 24 h. Then, 8 × 10^4^ cells C2C12 cells were carefully seeded onto each nanofiber disk. After 2 h of incubation at 37°C and 5% CO_2_, the volume of GM was completed to 500 μL/well. All experiments were performed in triplicate.

### Non-adherent cell counting

After 24 h of cell seeding, supernatants were carefully collected from the well and transferred to a falcon tube. The well was carefully washed with PBS, which was also transferred to the same falcon tube containing the supernatant. After centrifugation at 184 *g* for 5 min, the pellet was resuspended in 50 µL of fresh GM and the cells were counted using a Neubauer chamber.

### MTT assay for cell viability analysis

Cell viability was assessed using MTT assay (3-(4,5-dimethylthiazol-2-yl)-2,5-diphenyl tetrazolium bromide), according to the manufacturer’s instructions (Thermo Fisher Scientific). Briefly, C2C12 cells were seeded onto the nanofibers as previously described. After 2 and 7 days, GM was replaced with the MTT solution, and the samples were incubated for 2 h at 37°C and 5% CO_2_. Formazan crystals were then dissolved in 1 mL/well of isopropanol-acid (100 mL isopropanol:134 ul of hydrochloric acid). The solution was transferred to a 96-well plate in triplicate and absorbances were measured at 595 nm using a microplate reader (ELX800 device; BioTek, Winooski, USA).

### Cell morphology determined by SEM and F-actin staining

The morphology of the C2C12 cells cultivated onto CA and CA@A nanofibers was determined by i) SEM and ii) F-actin staining.

For SEM analysis, C2C12 cells were seeded onto CA and CA@A nanofibers and cultivated at 37°C and 5% CO_2_ in GM. After 2 and 7 days of culturing, samples were fixed in 2.5% glutaraldehyde for 6 h at RT. Samples were then rinsed with distilled water and gradually dehydrated in two increasing series of ethyl alcohol (35%, 50%, 70%, 85%, 95% and 100% for 15 min/bath). Samples were metalized with gold and visualized using a Quanta 200 FEG SEM (FEI, Hillsboro, USA).

For F-actin staining, C2C12 cells were seeded onto CA and CA@A nanofibers and cultivated at 37°C and 5% CO_2_ for 7 days in GM. After washing in PBS, cells were fixed in 3.7% formaldehyde for 15 min at RT. Samples were permeabilized in 0.1% Triton-X100 in PBS for 10 min at RT, washed with PBS, and incubated with 0.2 μg/mL Alexa Fluor 546 Phalloidin (Thermo Fisher) in PBS, for 30 min at RT. Next, cell nuclei were counterstained with DAPI (diluted to 1:1,000 in PBS) for 20 min at RT. Images were obtained in a Zeiss fluorescence microscope.

### RT-qPCR

1Cells were seeded onto each nanofiber in triplicate and cultivated for 7 days in GM only, or for 7 days in GM followed by an additional 7 days in DM. Both GM and DM were replaced by fresh medium every 2 days. All cells from the triplicate were then harvested in 1 mL TriReagent (Sigma-Aldrich) and the total RNA was isolated according to the manufacturer’s instructions. Next, 1 μg of each total RNA sample was converted into cDNA, following the instructions in the RevertAid H minus first strand cDNA synthesis kit (Thermo Fischer Scientific). GAPDH was used as a reference gene (AGG​TCG​GTG​TGA​ACG​GAT​TTG and TGT​AGA​CCA​TGT​AGT​TGA​GGT​CA). *MyoD*, *Myf5, MyoG* and *Desmin* were used as target genes with the following primers: for *MyoD* (GTG​GCA​GCG​AGC​ACT​ACA and GAC​ACA​GCC​GCA​CTC​TTC), for *Myf5* (GCA​AAG​ACC​CGT​GAC​TTC​AC and GCA​TGT​GGA​AAA​GTG​ATA), for *MyoG* (TGA​GAG​AGA​AGG​GGG​AGG​AG and CGG​TAT​CAT​CAG​CAC​AGG​AG) and for *Desmin* (GTG​GAG​CGT​GAC​AAC​CTG​AT and ATG​TTC​TTA​GCC​GCG​ATG​GT). RT-qPCR was performed using a Corbett 3,000 device (Qiagen, Helden, Germany), using 0.4–0.8 μM of each primer, 1 μL (diluted 1:10) of each cDNA, and 5 μL of iTaq universal SYBR Green Supermix (Bio-Rad, Hercules, USA), in a final volume of 10 μL. Reactions were performed as follows: 50°C for 2 min, 95°C for 2 min, followed by 45 cycles of 94°C for 15 s, 60°C–62°C for 15 s, and 72°C for 20 s. The dissociation step was performed at the end of the amplification step. Relative gene expression was determined using REST2009 software (based on the model by Pfaffl et al., 2002).

### Statistical analysis

All quantitative data are presented as means ± standard devi ations, and three repeated experiments were given. Statistical analysi s was performed using Student’s t-test or one-way analysis of variance followed by Fisher’s *post hoc* least-significant difference test for multiple comparisons. Differences were deemed significant at *p* < 0.05.

## Results

### Physicochemical, mechanical and morphological properties of the CA and CA@A nanofibers

In this work, the physicochemical and mechanical properties of the CA and CA@A nanofibers were performed using: i) the UV-vis spectroscopy, used specifically to confirm the annatto extract purity and its presence in the CA@A nanofiber; ii) the contact angle measurements, to determine the wettability properties of the nanofibers; and iii) the nanoscale dynamic mechanical analysis, to determine their mechanical properties.

**FIGURE 1:**
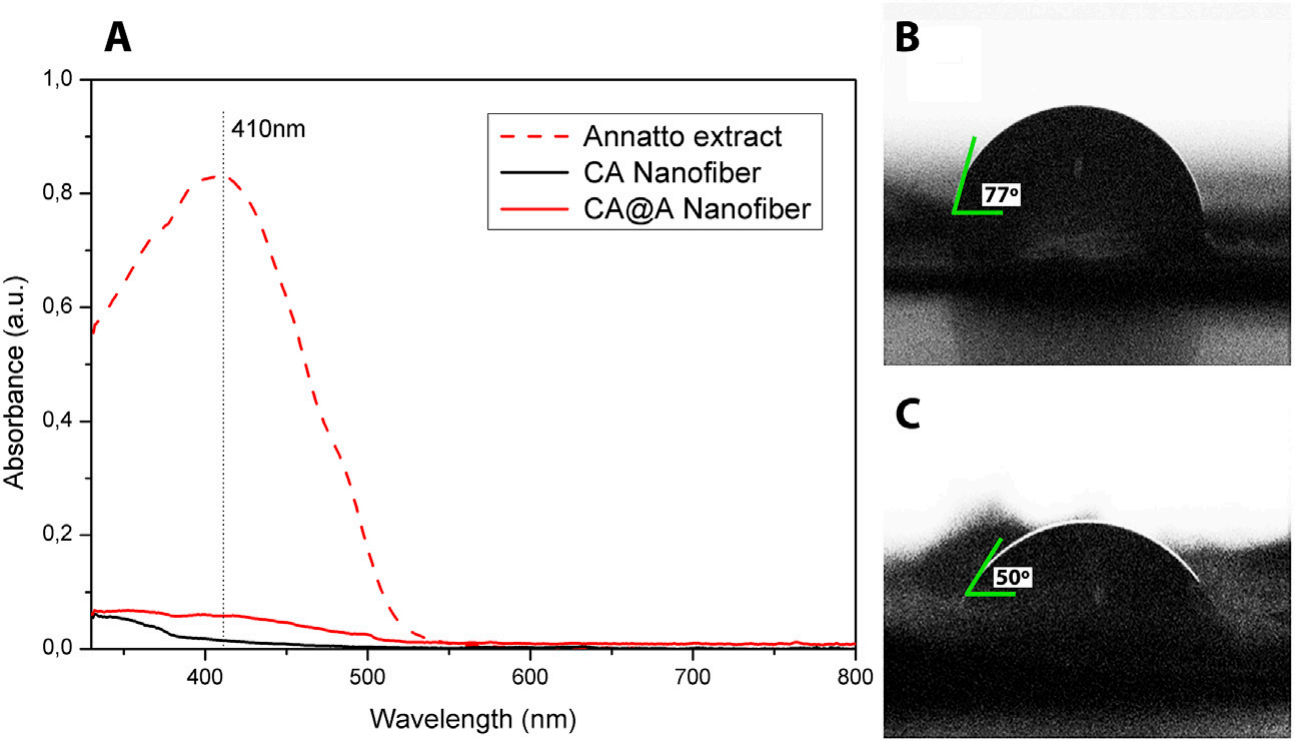
UV-absorption and water contact angle measurements. **(A)** UV-vis spectra of annatto extract, cellulose acetate nanofibers (CA), and cellulose acetate nanofibers with annatto extract (CA@A). Water contact angles of **(B)** cellulose acetate nanofiber (CA) and **(C)** cellulose acetate nanofiber impregnated with annatto extract (CA@A).

Nanofibers components were assessed by UV-vis spectroscopy (Figure 1A). The annatto extract spectrum revealed a strong and exclusive absorption band at 410 nm. No bands could be observed using CA nanofiber, while the CA@A sample revealed a low-intensity absorption band centered at 410 nm (Figure 1A).

Wettability property was assessed for CA and CA@A nanofibers by analyzing the contact angle (Figures 1B,C). The contact angle for the CA nanofiber was approximately 77° ± 3° (Figure 1B), while the addition of annatto to the CA nanofiber decreased the contact angle to 50° ± 3° (Figure 1C). Contact angles below 90° are characteristic of a hydrophilic property of the nanofibers.

**FIGURE 2:**
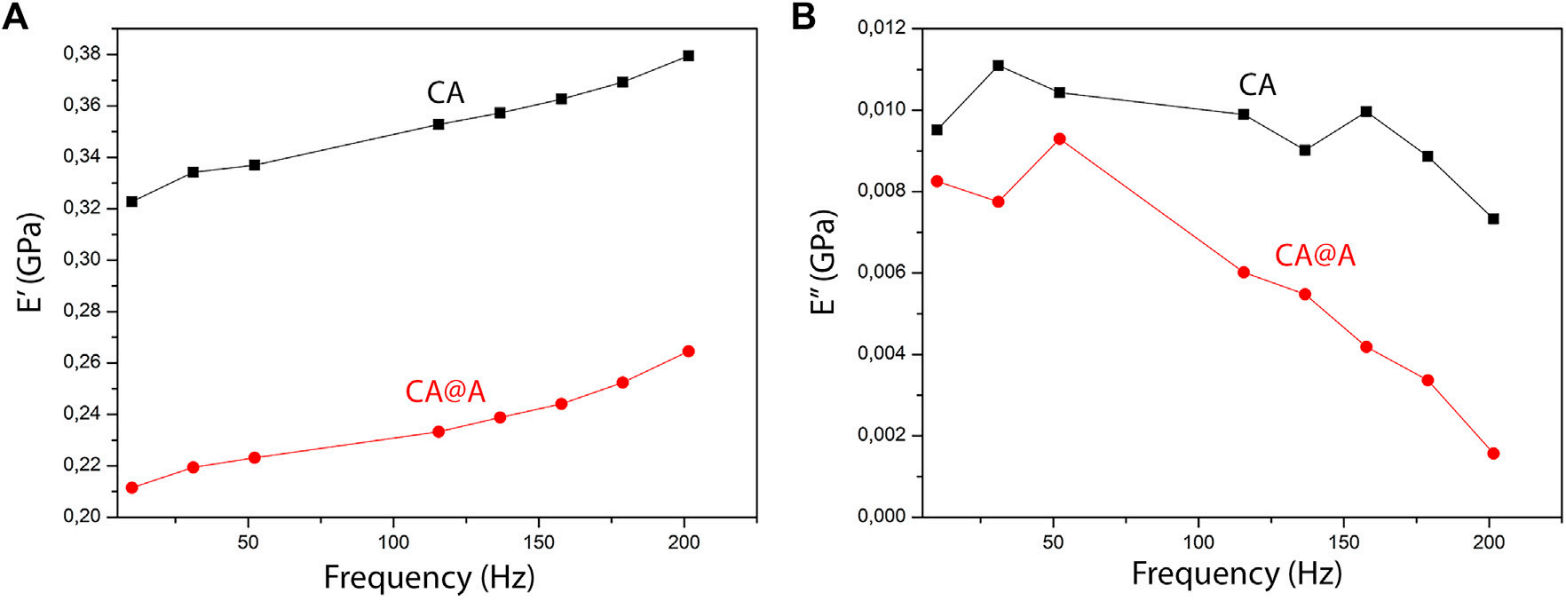
Dynamic mechanical analysis (DMA) of CA and CA@A nanofibers. **(A)** E′ (⌉) and **(B)** E” (⌉) curves for cellulose acetate (CA) and cellulose acetate nanofibers with annatto extract (CA@A) samples.

Mechanical properties of nanofibers at nanoscale were measured using Nano-DMA tests. Storage (E′) and loss (E″) modulus measurements for different frequencies are shown in Figure 2. Our results showed that the addition of annatto to the CA nanofibers decreased both E’ (Figure 2A) and E” (Figure 2B). At the frequency 10 Hz, E′ was 0.32277 GPa for the CA sample and 0.21148 GPa for the CA@A sample (Figure 2A), meaning that a reduction of 34% in terms of storage modules was achieved by adding annatto. For the loss modulus E” was 0.00952 GPa for the CA sample and 0.00826 GPa for the CA@A sample (Figure 2B), i.e., a reduction of 13.26%.

**FIGURE 3:**
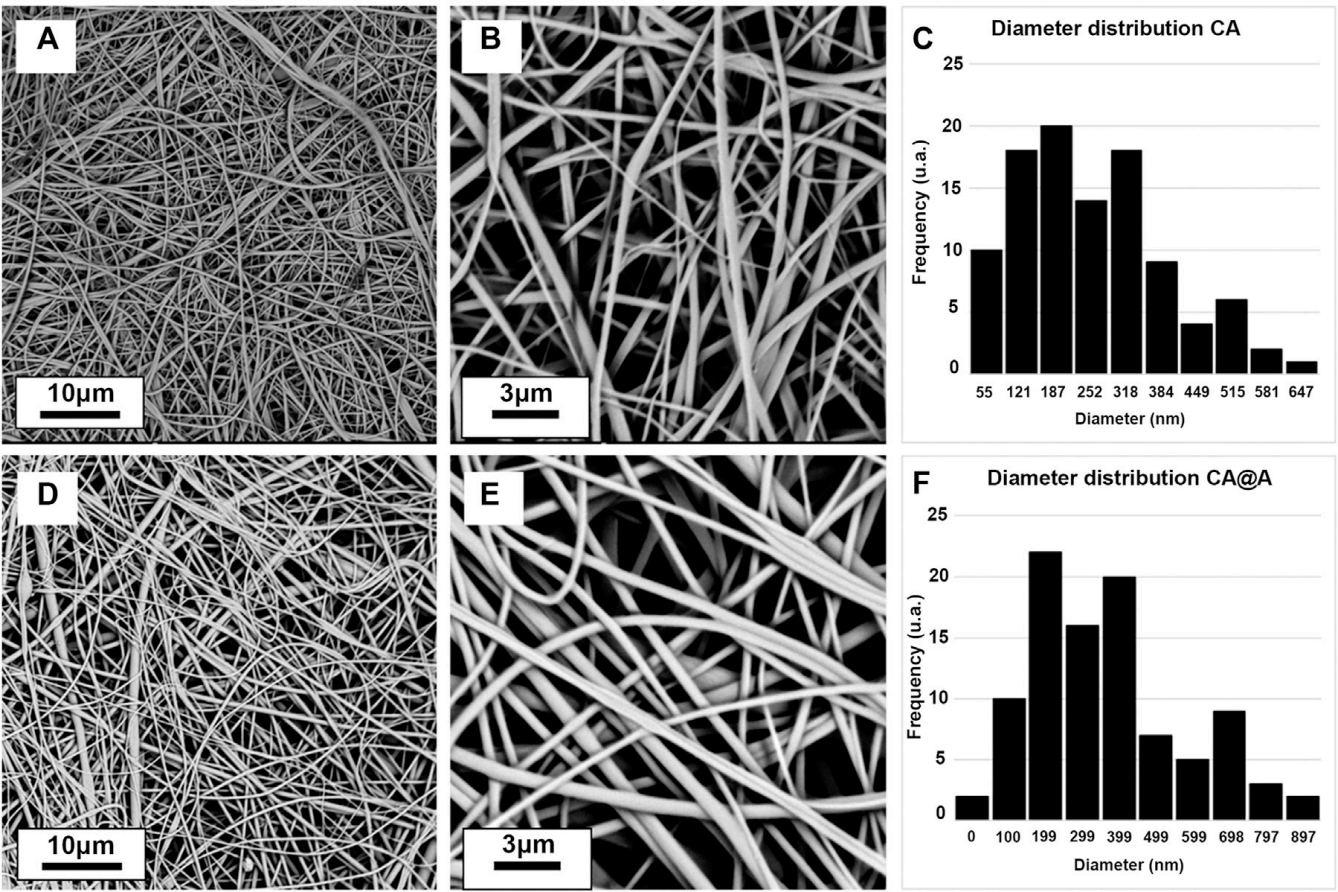
Scanning electron microscope (SEM) images of cellulose acetate (CA) and cellulose acetate annatto (CA@A) nanofibers at different magnifications and their size distribution. **(A, B)** Morphology of CA nanofibers with different magnifications. **(C)** Diameter distribution of CA nanofibers. **(D, E)** Morphology of CA@A nanofibers with different magnifications. **(F)** Diameter distribution of CA@A nanofibers.

We also obtained SEM images for both CA and CA@A nanofibers to allow their characterization based on the porous presence and the fiber diameters size (Figure 3). We found that both nanofibers presented smooth and relatively homogeneous porous mats and exhibited porous interconnectivity (Figures 3A,B for CA; Figures 3D,E for CA@A). We also analyzed fiber diameters and found that CA scaffolds present an average size of 284 ± 130 nm (Figure 3C), while the average size for CA@A was 420 ± 212 (Figure 3F).

### Both nanofibers allowed adherence and induce the viability of skeletal muscle cells

In this work, C2C12 myoblasts were used to evaluate the potential use of CA and CA@A nanofibers as scaffolds for the production of cultivated meat.

**FIGURE 4:**
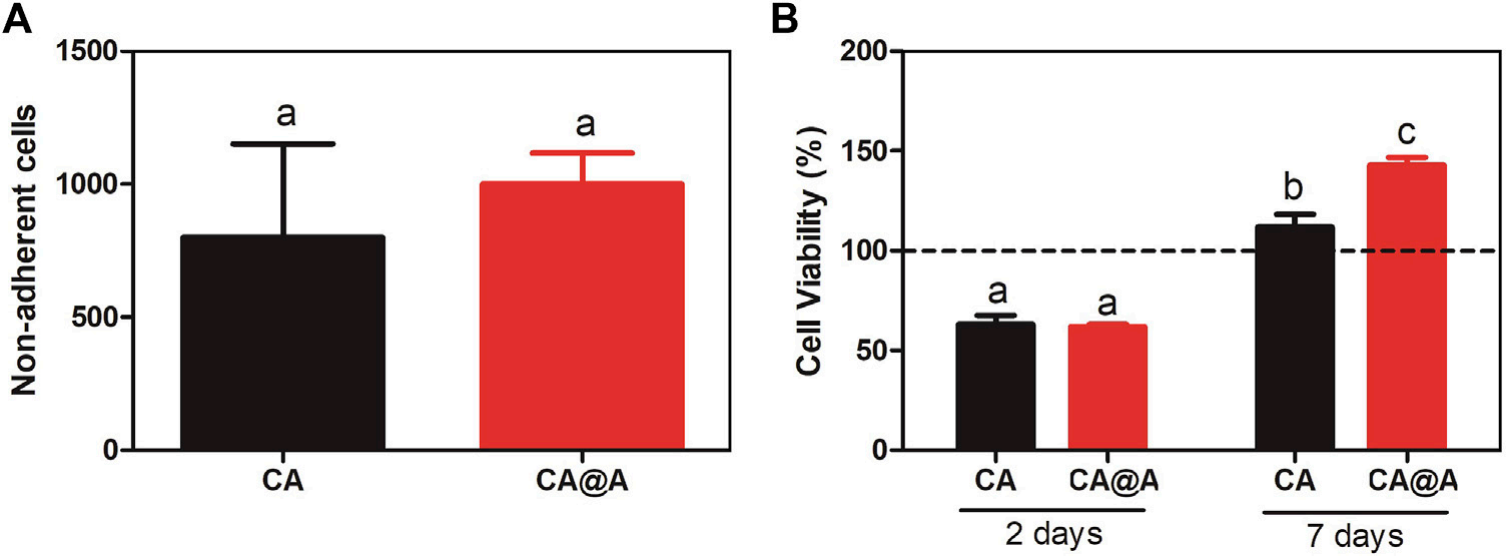
Initial analysis of cell-biomaterial adhesion and cell viability index. **(A)** C2C12 cell attachment to cellulose acetate (CA) and cellulose acetate nanofibers with annatto extract (CA@A) determined by cell supernatant counting after 24 h. **(B)** Graph representing % cell viability through the MTT assay of C2C12 myoblast cells incubated onto cellulose acetate (CA) and cellulose acetate nanofibers with annatto extract (CA@A) over 2 days and 7 days. The dotted line represents control, C2C12 plated on a monolayer for 2 and 7 days. Different letters demonstrate significant differences determined by a Student’s test (*p* < 0.05).

We first evaluated the capacities of these cells to attach to the CA and CA@A nanofibers, by counting non-adherent cells present in the medium after 24 h of cell culture (Figure 4A). From the ∼80,000 cells that were seeded onto each scaffold, ∼1,000 cells/well were unable to adhere to any of the substrates (Figure 4A). The rates of adherent cells were approximately 97.5% and 98% for cells cultivated onto CA and CA@A nanofibers, respectively. No significant difference was found between the cell number in the CA and CA@A nanofibers.

We also evaluated the viability index of C2C12 cells cultivated during 2 and 7 days onto CA and CA@A nanofibers, using MTT assay (Figure 4B).

After 2 days of culture, no difference in the viability indexes could be observed between CA and CA@A (Figure 4B). After 7 days of culture, an increase in the viability index could be observed for the cells cultivated onto both nanofibers, compared to the index observed after 2 days. Besides, the viability index of the cells cultivated onto the CA@A nanofiber was also found to be higher than for those cultivated onto the CA nanofiber, after 7 days of culture (Figure 4B).

Altogether these results suggest that both nanofibers allow great cellular attachment and also induce an increase in cell viability index over time. The presence of annatto in the nanofiber seems to confer an additional positive effect in muscle cell viability, compared to the ones cultivated onto the pure nanofiber.

### C2C12 cell morphology when cultivated onto both nanofibers

**FIGURE 5:**
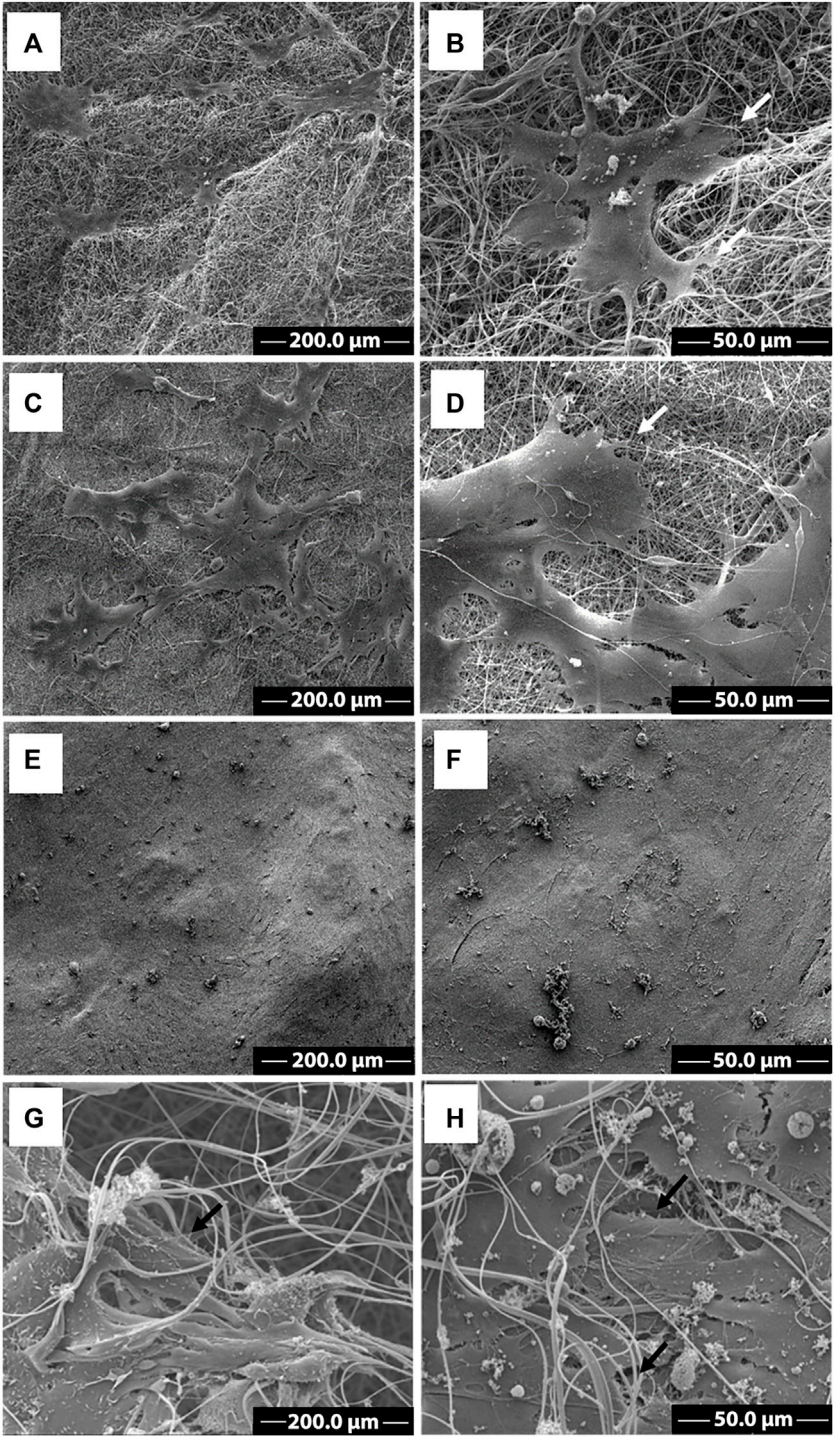
SEM images of C2C12 cells growth on CA and CA@A nanofibers. SEM images at different magnifications of C2C12 cells cultivated onto cellulose acetate nanofiber **(A, B)** and cellulose acetate nanofiber with annatto extract **(C, D)** after 2 days. SEM images at different magnifications of C2C12 cells cultivated onto cellulose acetate nanofiber **(E, F)** and cellulose acetate nanofiber with annatto extract **(G, H)** after 7 days. Scale bars indicate **(A,C,E, G)** 200 µm and **(B,D,F, H)** 50 µm. White arrows indicate cell-nanofiber adhesion points.

The morphology of C2C12 cells cultivated onto CA and CA@A nanofibers was analyzed using SEM images, after 2 and 7 days of culture in GM (Figure 5).

After 2 days of culture, myoblasts could colonize the surface of both CA (Figures 5A,B) and CA@A nanofibers (Figures 5C,D), but more cell groups could be observed in the CA@A (Figure 5C compared to 5A). The magnified image revealed that cells could already establish the first cell-cell contacts between them (Figures 5B,D). Cells were also found to produce extensions to establish links with both CA (Figure 5B, arrow) and CA@A (Figure 5D, arrow) nanofibers and showed a spindle-shaped morphology, like mononucleated myoblasts.

An exponential increase in cell density could be observed after 7 days of culture, allowing the covering of both nanofiber surfaces (Figures 5E–H). Almost all the cells stretched along the nanofibers and exhibited elongated morphology on both CA (Figure 5E) and CA@A nanofibers (Figure 5F). We could also observe myoblasts covered by nanofibers (Figures 5G,H), suggesting cell migration through the pores of the nanofibers.

**FIGURE 6:**
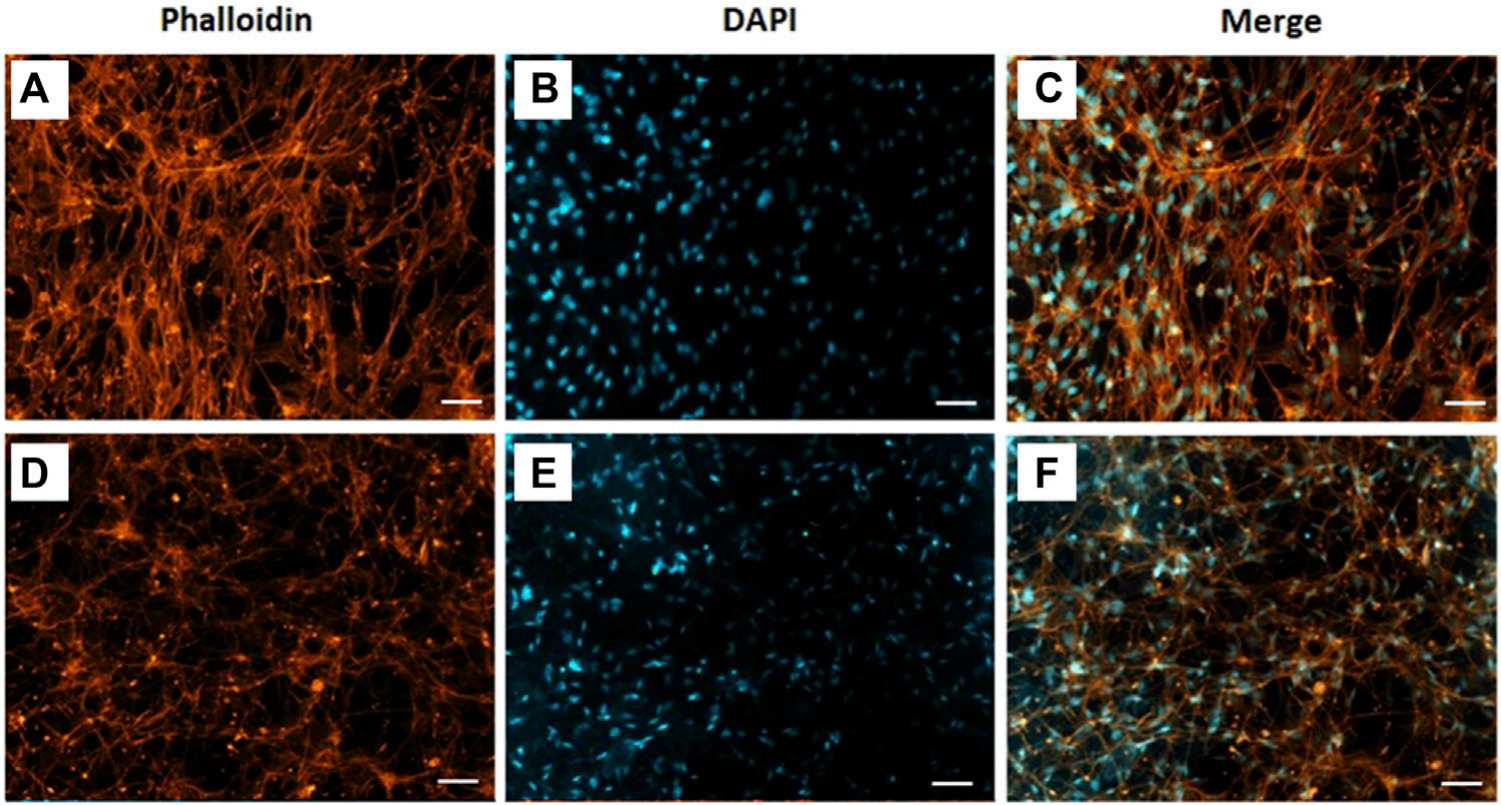
Phalloidin labelled F-actin (orange), DAPI nuclear staining (blue) and overlaid fluorescent image of C2C12 cellular components (merged) for CA **(A, B, C)** and CA@A **(D, E, F)**. Scale bar = 50 µm.

The morphology of C2C12 cells cultivated on CA and CA@A nanofibers was also assessed using fluorescence images from F-actin staining, a component of the cell cytoskeleton (Figure 6). Here, C2C12 cells were cultivated onto the nanofibers for 7 days, since the actin cytoskeleton is more easily resolved in higher density samples. The results showed that C2C12 cells cultivated onto the CA nanofiber were found to be more aligned and elongated (Figures 6A–C), compared to those cultivated onto the CA@A ones, which were found to be thinner and randomly distributed (Figures 6D–F).

### C2C12 cells differentiate when cultivated onto CA and CA@A nanofibers

**FIGURE 7:**
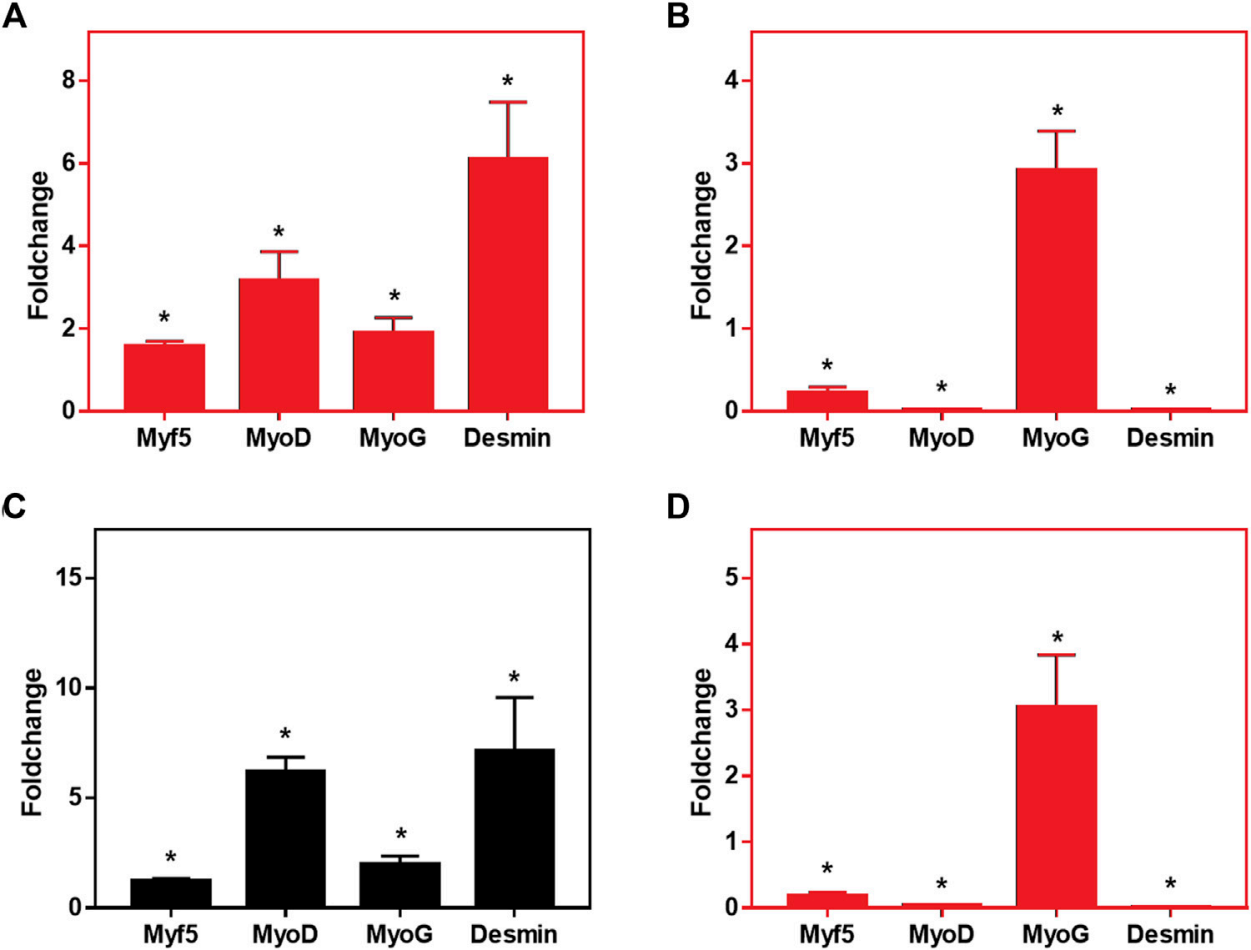
Relative expression levels of *MyoD*, *Myf5, MyoG* and *Desmin* in C2C12 cells cultured onto CA and CA@A nanofibers. **(A)** Relative gene expression analysis after 7 days of culture, comparing CA x CA@A. **(B)** Relative gene expression analysis after 14 days of culture, comparing CA x CA@A **(C)** Relative gene expression analysis in cells cultivated onto the CA nanofiber over time (7 days in GM x 7 days in GM followed by 7 days in DM). **(D)** Relative gene expression analysis in cells cultivated onto the CA@A nanofiber over time. Significative data obtained by REST2009 software, using *p* < 0.05.

We have also assessed whether C2C12 cells could reach differentiation when cultivated onto CA and CA@A nanofibers, by RT-qPCR (Figure 7). C2C12 cells were cultivated in GM during seven or in GM for 7 days followed by additional 7 days in DM (14 days), onto both nanofibers.

We first evaluated the relative gene expression by comparing the expression of the myogenic markers per nanofibers (CA x CA@A), at each analyzed day (7 and 14 days) (Figures 7A,B, respectively). After 7 days in culture, all myogenic markers were found to be upregulated in the cells cultivated onto the CA@A nanofiber, compared to the expression observed in the cells onto CA one (Figure 7A). After 14 days, however, which included 7 days of the cells cultivated in differentiation medium, relative gene expression analysis revealed that *Myf5*, *MyoD* and *Desmin* were downregulated in the cells cultivated onto the CA@A nanofiber, while *MyoG* was upregulated, all compared to the expression obtained on cells cultivated onto the CA nanofiber (Figure 7B).

We also evaluated the relative gene expression during cultivation (7 × 14 days) in each of the nanofibers (Figures 7C,D). Cells cultivated onto the AC nanofiber upregulated all myogenic markers at 14 days, compared to the expression at 7 days of culture (Figure 7C). When the same comparison was performed using the expression data of cells cultivated onto the AC@A nanofiber, however, again *Myf5*, *MyoD* and *Desmin* were found to be downregulated, while *MyoG* was upregulated at 14 days, all compared to the data obtained at 7 days of culture (Figure 7D).

## Discussion

In this work, we characterized the physicochemical and mechanical properties of nanofibers prepared by electrospun of cellulose acetate, as pure or containing annatto extract as a bioactive component, and the potential of applying these nanofibers as scaffolds to allow skeletal muscle cell growth and differentiation.

We first characterized the physicochemical, mechanical and morphological properties of both CA and CA@A nanofibers.

The UV spectrum of the pure annatto extract revealed a strong absorption band at 410 nm, which can be attributed to bixin and norbixin components of the annatto (Scotter, 2009; Giridhar, 2014; Rahmalia et al., 2015). No peaks attributed to additional annatto compounds were observed (Calogero et al., 2015; Pinzón-Garcia et al., 2016). The presence of annatto extract in the CA@A nanofiber was confirmed by a low-intensity absorption band centered at 410 nm.

Contact angle measurements allowed us to evaluate the wettability properties of the CA and CA@A nanofibers. Surface wettability is extremely important for cell adhesion, as hydrophilicity is the intrinsic property of the natural extracellular matrix (Menzies and Jones, 2010). Our results showed that both nanofibers exhibit hydrophilic properties. The enhancement in the wettability observed for the CA@A nanofiber compared to the CA nanofiber is associated with the hydrophilic nature of the bixin and norbixin molecules present in the annatto extract.

Mechanical properties play an important role in cell adhesion, differentiation, morphology and migration. In order to evaluate the effect of annatto on the stiffness of the nanofibers, we performed the Nano-DMA test. Our results revealed that the addition of annatto to cellulose acetate decreased the stiffness of the obtained nanofibers. Similar results were reported in previous studies showing a reduction in polymer stiffness as a result of adding eugenol, ginger, cinnamon, guarana, and rosemary extract (Bonilla et al., 2018; Ke et al., 2019; Moeini et al., 2022). Furthermore, the nanofiber stiffnesses obtained in our work (323 MPa and 211 MPa) presented comparable values to the electrospun matrices developed for muscle tissue engineering (Cooper et al., 2010; Riccotti et al., 2012; Luo et al., 2018; Jekins and Little, 2019).

Skeletal muscle ECM is a complex meshwork consisting of collagens, glycoproteins, proteoglycans, and elastin. Collagen fibrils in the skeletal muscle ECM vary in diameter from 30 to 300 nm (Ushiko, 2002), while elastin fibers are about 100 nm thick (Gasser, 2017). Morphological analysis revealed by SEM images showed that both CA and CA@A nanofibers present a smooth and homogeneous porous mat, exhibiting porous interconnectivity. This is considered an important property of a material to be used as scaffold for cell growth, since the porous presence allows cell migration and the colonization of the interior of the scaffold (Post et al., 2020). Porous nature might also allow vascularization as well as the formation of multiple layers of cells, both crucial processes for establishing a tissue-like construct (Gurdon et al., 1993). Besides that, Csapo et al. (2020) investigated the manner in which myoblasts detect and respond to fiber diameter differences and found that increased fiber diameters (from 335 ± 154 nm to 3,013 ± 531 nm) were able to induce myoblast proliferation and differentiation, as well as fusion into mature myotubes, indicating the ability of cells to respond to fiber topography. Our morphological analysis also showed that there was an increase in the CA@A diameter fibers when compared to CA nanofibers.

We next evaluated the biocompatibility of these nanofibers to support skeletal muscle cell growth and differentiation. Myoblasts from the C2C12 immortalized cell lineage were used in this work since they are easy to manipulate and are an excellent model to test the possibility of use of these nanofibers as scaffolds in muscle tissue engineering. In the presence of serum, C2C12 myoblasts are induced to proliferate. When these cells start making contact with each other, or when serum is removed from the medium, C2C12 cells initiate the differentiation program, meaning that these cells suffer growth arrest, elongate and fuse to each order to form a multinucleated myofiber (Bruyère et al., 2019).

Both nanofibers revealed significant capacity of cell attachment, since we found only ∼2% of the cells free in the medium after 24 h of plating. Besides attachment, cell viability analysis revealed that C2C12 cells were similarly viable after 2 days in culture onto both nanofibers. SEM images corroborated with this finding and allowed the observation of the first cell-cell and cell-nanofibers contacts. Cells tend to connect to each other and to sense the environment in which they were placed, showing the ability to recognize and interact with that milieu (Wijnhoven et al., 2020) and long-term behavior is highly dependent of the cell shape and cytoskeletal organization that are often initiated during the minutes to hours following adhesion (Cretel et al., 2008).

Both nanofibers induced an increase in cell viability index after 7 days of culture, suggesting that their large surface area is beneficial for long-duration cell culture. Again, SEM images corroborated with this data. However, the presence of annatto improved the viability index of C2C12 cells after 7 days in culture compared to pure CA nanofibers. The improvement in cell viability in CA@A nanofibers might occur due to i) the increased hydrophilicity of the nanofiber due to the addition of annatto (Golizadeh et al., 2019; Jenkins and Little, 2019; Zan et al., 2020) and ii) the presence of the antioxidant components in the annatto extract (Naranjo-Durán et al., 2021).

We have also investigated whether cell seeding and culturing onto CA and CA@A nanofibers would interfere with the progression of the differentiation process of these cells during time. We first evaluated cell shape by staining the F-actin component of the cytoskeleton after 7 days of culture. C2C12 cells plated onto CA nanofiber were found to be more aligned and elongated, while the same cells showed to be thinner and randomly distributed when cultivated onto CA@A.

To better assess the cell differentiation stage, we evaluated the gene expression pattern of the main myogenic markers in C2C12 cells cultivated onto CA and CA@A nanofibers. Here we investigated the expression of *MyoD* and *Myf5,* which are markers expressed first during the myogenesis process, being more related to the proliferation stage of these cells; and *MyoG* and *Desmin*, which are related to the cell fusion stage and is necessary to form multinucleated myotubes (Chal & Pourquié, 2017).

We found that cells cultivated onto CA@A nanofibers upregulate the expression of myogenic markers already after 7 days in culture, even cultivating these cells only with GM, suggesting that the annatto has an impact in inducing C2C12 cell differentiation. After 14 days of culture in GM, we could only observe the induction of *MyoG* expression, being all the other markers found as downregulated. This result suggested that, despite showing an important effect on cell differentiation at the beginning, the annatto might be interfering with the phenotype of these cells during time, since we could not observe an impact in all late myogenic markers. These results were corroborated with the analysis performed using the data obtained during time (7 × 14 days): the differentiation progress could be observed on cells cultivated onto the CA nanofiber over time, since all myogenic markers were found to be upregulated at 14 days, compared to their expression at 7 days, while AC@A allowed the upregulation of only *MyoG* over time. Altogether, these results suggested that the expression of myogenic markers are favored in cells cultivated onto CA nanofibers, while the annatto interfere with the myogenic differentiation of these cells.

The greater differentiation of C2C12 in pure nanofibers compared to nanofibers containing annatto can be associated with scaffold mechanical and morphological properties. In the present work we have shown that pure nanofiber has higher stiffness and smaller fiber diameter, which greatly contributed to C2C12 cellular differentiation. Our result is compatible with previous works showing more differentiated myoblasts on nanofibers with a smaller diameter and a higher stiffness (Choi et al., 2008; Ku et al., 2012; Luo et al., 2018). Altogether, these results suggested that the expression of myogenic markers are favored in cells cultivated onto CA nanofibers, while the annatto interfere with the myogenic differentiation of these cells.

Here, we demonstrated the adhesion, proliferation, and differentiation of muscle cells in the cellulose acetate nanofiber as a preliminary stage towards its application in cultured meat production. To obtain cultured meat, however, it is still crucial to study the interaction between the CA scaffolds and cells from agriculturally relevant species such as beef, pork, poultry and seafood.

## Data Availability

The original contributions presented in the study are included in the article/supplementary material, further inquiries can be directed to the corresponding authors.
